# CRISPR/Cas9 Genome Editing to Disable the Latent HIV-1 Provirus

**DOI:** 10.3389/fmicb.2018.03107

**Published:** 2018-12-14

**Authors:** Amanda R. Panfil, James A. London, Patrick L. Green, Kristine E. Yoder

**Affiliations:** ^1^Department of Veterinary Biosciences, College of Veterinary Medicine, The Ohio State University, Columbus, OH, United States; ^2^Center for Retrovirus Research, The Ohio State University, Columbus, OH, United States; ^3^Department of Cancer Biology and Genetics, College of Medicine, The Ohio State University, Columbus, OH, United States

**Keywords:** HIV-1, latency, retrovirus, CRISPR/Cas9, genome editing, anti-retroviral therapy

## Abstract

HIV-1 infection can be successfully controlled with anti-retroviral therapy (ART), but is not cured. A reservoir of cells harboring transcriptionally silent integrated provirus is able to reestablish replicating infection if ART is stopped. Latently HIV-1 infected cells are rare, but may persist for decades. Several novel strategies have been proposed to reduce the latent reservoir, including DNA sequence targeted CRISPR/Cas9 genome editing of the HIV-1 provirus. A significant challenge to genome editing is the sequence diversity of HIV-1 quasispecies present in patients. The high level of quasispecies diversity will require targeting of multiple sites in the viral genome and personalized engineering of a CRISPR/Cas9 regimen. The challenges of CRISPR/Cas9 delivery to the rare latently infected cells and quasispecies sequence diversity suggest that effective genome editing of every provirus is unlikely. However, recent evidence from post-treatment controllers, patients with controlled HIV-1 viral burden following interruption of ART, suggests a correlation between a reduced number of intact proviral sequences and control of the virus. The possibility of reducing the intact proviral sequences in patients by a genome editing technology remains intriguing, but requires significant advances in delivery to infected cells and identification of effective target sites.

## Introduction

**FIGURE 1:**
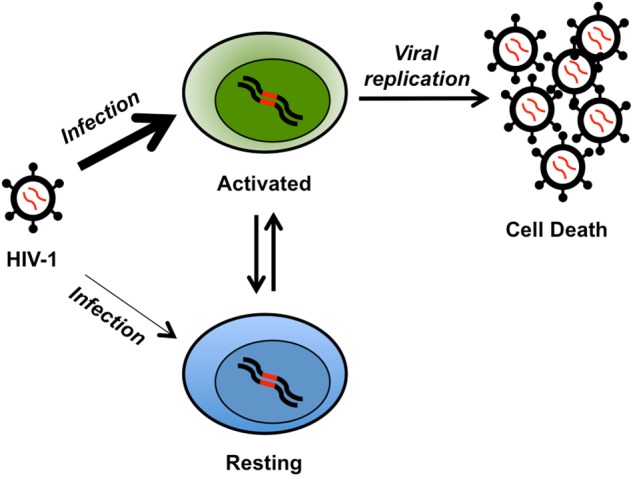
Cartoon of latent reservoir formation. The HIV-1 virus readily infects (thick arrow) activated CD4^+^ T cells (green, integrated provirus in red). HIV-1 replication in activated T cells typically leads to cell death. However, a fraction of these cells may become resting memory T cells (blue) and part of the latent reservoir. A perhaps less frequent (thin arrow) alternative mechanism for latency is the infection of resting naïve CD4^+^ T cells (blue). The latently infected resting naive or memory CD4^+^ T cells may become activated, leading to viral replication and cell death.

Retrovirus HIV-1 reverse transcribes a viral RNA genome to a linear double-stranded complementary DNA (Coffin et al., 1997). The genome integrates into the host genome, termed a provirus. The provirus may become latent, defined by lack of transcription (Van Lint et al., 2013). The mechanisms that drive latency are not entirely known but include transcription interference, repressive histone modifications, and absence of the HIV-1 Tat protein (reviewed in Donahue and Wainberg, 2013). Long-lived memory CD4^+^ T cells harbor latent proviruses and other cell types may also be part of the latent HIV-1 reservoir (Figure 1; Chun et al., 1995, 1997; Murray et al., 2016). Anti-retroviral therapy (ART) suppresses HIV-1 replication, but does not affect latently infected cells. The number of cells with an inducible latent provirus is estimated to be ∼1 in 10^6^ resting CD4^+^ T cells (Siliciano et al., 2003; Crooks et al., 2015). Without transcription of viral genes, such as *env*, there are no obvious markers of latently infected cells making them difficult to identify (Badia et al., 2018). Importantly, intact latent proviral genomes remain capable of reactivation and replication and HIV-1 viral load increases within weeks of stopping ART (Li et al., 2016; Colby et al., 2018; Wen et al., 2018).

## Eliminating the Hiv-1 Latent Reservoir

Several strategies have been proposed to purge the latent reservoir. One is to activate the provirus with histone deacetylase (HDAC) inhibitors (Shirakawa et al., 2013; Manson McManamy et al., 2014; Walker-Sperling et al., 2016). Activated viral transcription and replication should induce cell death. Unfortunately, HDAC inhibitors have not eliminated the latent reservoir (Rasmussen and Lewin, 2016; Kim et al., 2018). The drugs induce HIV-1 transcription, but the cells do not die, likely due to cellular mechanisms promoting survival (Rasmussen and Lewin, 2016; Kim et al., 2018).

Genome editing with zinc finger nucleases (ZFNs) targeting the *CCR5* gene in hematopoietic stem cells (HSCs) has entered clinicial trials (Tebas et al., 2014). ZFNs are a fusion of DNA sequence specific zinc finger domains and a Fok1 nuclease domain. A pair of ZFNs generate a DNA double strand break (DSB) at a sequence specific site. DSBs are commonly repaired by the error prone non-homologous end joining (NHEJ) pathway resulting in short insertions or deletions (indels). NHEJ repair can disrupt the reading frame of a targeted gene leading to a null phenotype. The human *CCR5* gene was chosen because HIV-1 must bind CD4 and either CCR5 or CXCR4 to infect cells. Multiply exposed uninfected individuals encode a homozygous 32 bp deletion in the *CCR5* gene leading to lack of cell surface expression (Liu et al., 1996). This suggested that abrogation of *CCR5* expression would be tolerated and prevent infection. In this therapeutic approach, a ZFN pair targeting the *CCR5* gene is added to patient HSCs *ex vivo*. Edited cells are reinfused to the patient. ZFN editing of both *CCR5* alleles in HSCs should generate CD4^+^ T cells resistant to HIV-1. Limited success has been achieved, but not a cure (Tebas et al., 2014). Additional genome editing strategies have been proposed to delete or disable the HIV-1 provirus, including other ZFNs, transcription activator-like effector nucleases (TALENs), or engineered endonucleases (Aubert et al., 2011; Qu et al., 2013; Ebina et al., 2015; De Silva Feelixge et al., 2016; Karpinski et al., 2016). However, ZFNs and other endonucleases require significant engineering to target specific DNA sequences and edit only a single site.

**FIGURE 2:**
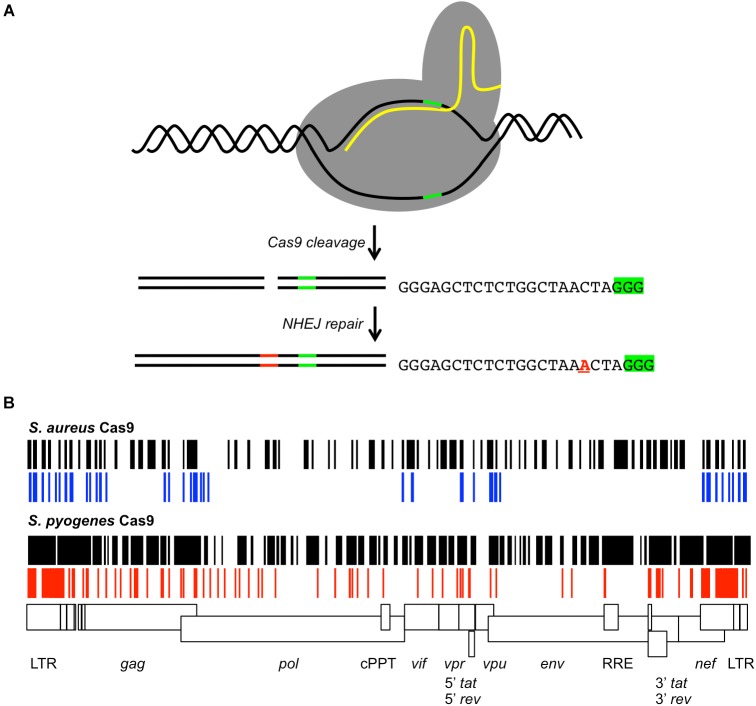
CRISPR/Cas9 gRNAs targeting the HIV-1 provirus. **(A)** Cartoon of Cas9 cleavage of target DNA. The Cas9 protein (gray) binds the target DNA (black lines) and gRNA (yellow line). The PAM signal (green line) is adjacent to the gRNA homology region. Cas9 generates a DSB 3 bp 5′ of the PAM. DNA repair by NHEJ may insert or delete nucleotides (red lines) at the cleavage site. As an example, the sequence of a gRNA targeting the HIV-1 TAR element is shown with the PAM signal 5′-GGG-3′ (green highlight). The observed repair products included insertion of a single nucleotide at the cleavage site (red A) (Yoder and Bundschuh, 2016). **(B)** The possible *S. aureus* or *S. pyogenes* gRNAs throughout the proviral genome of the laboratory strain HXB2 for Cas9 are shown (black) relative to a scale map of the HIV-1 genome. Previously reported *S. aureus* gRNAs are shown in blue, *S. pyogenes* gRNAs in red. Features of the HIV-1 genome are indicated by boxes. Differences between the possible gRNAs and reported gRNAs are due to strain differences.

The most adaptable strategy recently applied to proviral genome editing is CRISPR/Cas9. This genome editor consists of a Cas9 endonuclease from bacteria that generates a DSB at a sequence specific site (Cho et al., 2013; Cong et al., 2013). The DSB is directed by a 20 nucleotide guide RNA (gRNA) fused to a scaffold RNA (Figure 2A). Cas9 in complex with the fusion RNA will recognize the target DNA sequence with a 3′ protospacer adjacent motif (PAM) that is not encoded in the gRNA. *Streptococcus pyogenes* Cas9 (SpCas9) recognizes a 5′-NGG-3′ PAM and *S. aureus* Cas9 (SaCas9) PAM is 5′-NNGRRT-3′. The target DNA is cleaved 3 base pairs (bp) away from the PAM within the gRNA target sequence. Cas9 induced DSBs are often repaired by NHEJ. CRISPR/Cas9 gRNA engineering to specific DNA sequences is facile, simplifying designer therapy. In addition, CRISPR/Cas9 allows targeting of multiple sites simultaneously with gRNAs. The choice of SpCas9 or SaCas9 is based on the size limit of the delivery technology, as the *SpCas9* gene is 4.1 kb and *SaCas9* is 3.1 kb, as well as the target site PAM. There are more possible SpCas9 target sites throughout the HIV-1 genome compared to SaCas9 (Figure 2B). Although CRISPR/Cas9 genome editing has been suggested as a method for disabling HIV-1 proviral genomes, empirical validation of the approach has assayed editing in latent cell lines and replicating HIV-1 infection in cell lines. Analysis of CRISPR/Cas9 proviral editing in a replicating HIV-1 infection allowed the identification of mutations leading to resistance (Wang et al., 2016b,d; Yoder and Bundschuh, 2016). Few reports have employed primary human CD4^+^ T cells (Liao et al., 2015; Kaminski et al., 2016b).

## Crispr/Cas9 Genome Editing Approaches to Disable HIV-1

CRISPR/Cas9 genome editing of the provirus has employed two strategies. One exciting concept targeted the long terminal repeats (LTRs) present at each end of the provirus (Ebina et al., 2013; Dampier et al., 2014; Hu et al., 2014; Kaminski et al., 2016a,b; Yin C. et al., 2016; Yin C. et al., 2017; Bella et al., 2018). The LTRs are perfect repeats. Generating DSBs in both LTRs with a single gRNA could conceivably delete the interior of the provirus leaving a single LTR remnant (Figure 2B). Some studies reported that deletion of the ∼8.5 kb provirus occurred, but other groups did not see this result (Yoder and Bundschuh, 2016; Mefferd et al., 2018). This apparent discrepancy can be reconciled by a model where large CRISPR/Cas9 generated deletions occur at relatively low frequencies (Canver et al., 2014; Byrne et al., 2015). Genomic deletions of similar size were observed with frequency <15% in human induced pluripotent stem cells or <25% in murine erythroleukemia cells (Canver et al., 2014; Byrne et al., 2015). CRISPR gRNAs targeting the LTRs may delete the provirus, but with low efficiency.

Other groups disabled the provirus via NHEJ indels (Liao et al., 2015; Zhu et al., 2015; Ueda et al., 2016; Wang et al., 2016b,a,d; Yoder and Bundschuh, 2016; Mefferd et al., 2018; Ophinni et al., 2018; Roychoudhury et al., 2018). Sites throughout the HIV-1 genome have been targeted with SpCas9 and SaCas9, although the empirically tested gRNAs are not an exhaustive list (Figure 2B). The gRNAs display variable efficiencies in reducing HIV-1 expression or replication. There is no mechanism to predict the efficiency of the gRNAs. HIV-1 is known to develop resistance to drug monotherapy and a single gRNA was no exception (Ueda et al., 2016; Wang et al., 2016b,d; Yoder and Bundschuh, 2016). HIV-1 strains with resistance to a single gRNA were shown to develop by either mutagenic reverse transcription or NHEJ. A single indel at the repair junction proved sufficient to prevent further CRISPR/Cas9 editing.

The time to develop resistance varied between CRISPR gRNAs and suggested differences in DSB/indel genetic fitness throughout the viral genome. Strains resistant to gRNAs targeting non-coding and non-structured regions of the LTR developed the fastest, suggesting these regions are genetically robust and tolerant of indels (Wang et al., 2016a,b,d; Yoder and Bundschuh, 2016). However, non-protein coding regions that form the important TAR RNA stem loop are empirically better targets (Yoder and Bundschuh, 2016; Mefferd et al., 2018). There are several possible SpCas9 gRNA targets within TAR displaying variable efficiencies; some TAR gRNAs are able to delay HIV-1 replication for several days and one TAR gRNA appeared to eliminate replication (Yoder and Bundschuh, 2016; Mefferd et al., 2018). Many studies of HIV-1 genome editing by CRISPR/Cas9 did evaluate resistance. While it is logical that protein coding regions do not tolerate single bp indels and might be the best targets for genome editing, there is evidence that 3 bp indels can form and preserve the protein reading frame (Wang et al., 2016a; Yoder and Bundschuh, 2016). Such genetic changes might not disrupt a viral protein to the point of a null phenotype and prevent HIV-1 replication.

More recently the provirus was targeted with multiple gRNAs simultaneously (Wang et al., 2016a; Yin C. et al., 2017; Bella et al., 2018; Ophinni et al., 2018; Roychoudhury et al., 2018; Wang Q. et al., 2018). This approach has abrogated replication of HIV-1 laboratory strains in cell lines. However, it has not been possible to deliver multiple gRNAs with Cas9 in a single vector, which is a significant limitation. In bacteria, CRISPR gRNAs are multiplexed and separated by direct repeat sequences, which are cleaved to form a complex with Cas9. Single transcript multiplexing of gRNAs in mammalian cells requires an additional system for processing to single gRNAs, such as the *P. aeruginosa* Csy4 RNA nuclease (Sternberg et al., 2012; Kurata et al., 2018). Current strategies for multiplexing gRNAs in mammalian cells require transfection of multiple plasmids, transduction of multiple vectors, or expression from multiple independent RNA pol III promoters. Two to seven gRNAs driven by U6, H1, and/or 7SK promoters in a single CRISPR/Cas9 delivery vector have been described (Kabadi et al., 2014; Sakuma et al., 2014; Merienne et al., 2017; Petris et al., 2017). Delivery of CRISPR/Cas9 with relatively few gRNAs in a single vector is a limitation that will have to be overcome.

## Challenges of Genome Editing the Hiv-1 Provirus

Multiplexing of CRISPR gRNAs will likely be required for effective HIV-1 genome editing, but it is not clear how many or which proviral sites should be targeted. One criteria is the sequence conservation of the target site. The site must also be efficiently cleaved by Cas9. However, these criteria are not sufficient. For example, the sequence surrounding the HIV-1 TATA box, required for all HIV-1 transcription, is well conserved among subtype B isolates (Yoder and Bundschuh, 2016). This region is efficiently cleaved by Cas9 and repaired with indels. HIV-1 resistance to a gRNA targeting this conserved site develops rapidly indicating that the HIV-1 TATA box is not genetically fragile and not a useful target (Yoder and Bundschuh, 2016). Other studies have shown that combination of strongly suppressive gRNAs is superior to combinations including weakly suppressive gRNAs (Lebbink et al., 2017). There has been no large scale direct comparison of CRISPR gRNAs targeting sites throughout the HIV-1 provirus. CRISPR/Cas9 gRNA targets highly efficient for preventing HIV-1 replication have been identified, but an empirically determined DSB genetic fitness map of the HIV-1 provirus would indicate the most genetically fragile sites and the best targets for genome editing (Wang et al., 2016a; Lebbink et al., 2017; Mefferd et al., 2018; Yoder, 2018).

The number of CRISPR/Cas9 gRNAs required to edit the HIV-1 provirus in patients will depend on the quasispecies sequence diversity. Two groups have investigated potential CRISPR gRNA targets in HIV-1 and their sequence conservation within patient quasispecies (Dampier et al., 2014; Roychoudhury et al., 2018). The first study sequenced HIV-1 quasispecies from peripheral blood mononuclear cells (PBMCs) of 6 patients, including two time points 11 months apart from 2 patients (Dampier et al., 2014). Of these 8 samples, a panel of ≤10 gRNAs targeting the LTR of all quasispecies could be engineered for 4 samples. Interestingly, a gRNA panel targeting all quasispecies of one patient could not be designed for the first time point, but was possible at the second. In a second study, CRISPR gRNAs targeting the *pol* gene were compared to deep sequencing of HIV-1 quasispecies from PBMCs of 4 patients (Roychoudhury et al., 2018). The gRNA targeted sites displayed <87% sequence conservation within each patient. The authors caution that rare quasispecies not efficiently cleaved may prevent a functional cure. Indeed, if targets are cleaved and repaired without a frameshift (±3 bp indels preserving the reading frame), the provirus may remain competent for replication (Yoder and Bundschuh, 2016). Together, these studies suggest that patient samples must be sequenced to identify quasispecies targets immediately prior to design of a CRISPR regimen consisting of several gRNAs. However, comparison of HIV-1 proviral sequences from PBMCs and lymph nodes or cerebral spinal fluid (CSF) suggests that these quasispecies populations are distinct (Haddad et al., 2000; Fourati et al., 2014). Thus, the feasibility of effectively purging all latent HIV-1 proviruses with sequence specific genome editing is unclear.

The goal of eliminating all HIV-1 replication competent proviruses is based on the notion that a single provirus can lead to viral rebound. However, there is recent evidence from post-treatment controllers (PTCs) that this assumption is not necessarily true (Saez-Cirion et al., 2013; Sharaf et al., 2018). Post-treatment control has been defined as an HIV-1 viral load of <400 copies/ml for at least 24 weeks following ART interruption (ATI) (Sharaf et al., 2018). PTCs are rare, but have been identified in several patient groups as well as among patients who began ART in either acute or chronic phase of infection (Perkins et al., 2017). A recent study comparing the proviral sequences of PTCs and non-controllers (NCs) found that total number of proviruses is lower in PTCs prior to ATI (Sharaf et al., 2018). This “total” number of proviruses included both intact and defective proviruses, where defective proviruses are always a significant majority (Bruner et al., 2016; Pollack et al., 2017; Sharaf et al., 2018). Intuitively, only the intact proviral sequences would contribute to viral rebound and a NC phenotype; indeed, the number of observed intact proviral sequences was less in PTCs (PTCs: 0.04/10^6^ PBMCs; NC: 0.28/10^6^ PBMCs, *p* < 0.05) (Sharaf et al., 2018). Interestingly, the number of proviruses did not increase in PTCs after ATI, unlike in NCs. It is conceivable that a genome editing based reduction of the intact replication competent proviral load could lead to a PTC phenotype. In this scenario patients harbor defective proviral sequences, but do not require ART. A counter argument is that defective proviruses express viral mRNAs and antigens recognized by the immune system (Pollack et al., 2017; Sharaf et al., 2018). Depending on the gRNA target site, CRISPR/Cas9 edited proviruses may express HIV-1 RNAs. The relative accumulation of cell associated HIV-1 RNAs are known to predict the timing to viral rebound following ATI (Li et al., 2016). Whether significantly decreasing the intact proviral reservoir will be sufficient for a PTC phenotype is unknown, but may become apparent with additional study of proviral genomes present in PTCs before and during ATI. Similarly, the effects of increasing the defective proviral reservoir on patient biology are difficult to predict.

A significant question regarding genome editing as a treatment strategy for HIV-1, as well as many other diseases, concerns the delivery to target cells. Among the hurdles to overcome are the scarcity of cells harboring a latent HIV-1 provirus and the lack of a unique cell surface marker (Siliciano et al., 2003; Crooks et al., 2015; Badia et al., 2018). If a latency specific cell surface marker is identified, then it may be possible to deliver CRISPR/Cas9 via viral vectors. While adeno-associated vector particles are attractive due to their low immunogenicity, they are not known to infect CD4+ T cells, the major reservoir cell type. Lentiviral vector particles may be an attractive delivery method since they are readily pseudotyped and may encode ∼8.5 kb of genetic cargo. Alternatively, HSCs could be engineered with an inducible CRISPR/Cas9 *ex vivo* to populate the immune system with anti-retroviral defense. Indeed, CRISPR/Cas9 introduced to cells before HIV-1 infection was shown to protect cells (Liao et al., 2015). While transfection of plasmids or nucleofection of purified complexes of recombinant Cas9 protein complexed with synthetic gRNA and scaffold RNA are efficient delivery mechanisms in cell culture, it is difficult to imagine these methods translating to *in vivo* delivery. Alternative delivery technologies, such as Sendai virus vectors, are in development (Park et al., 2016, reviewed in Lino et al., 2018).

The safety of any genome editing technology is a concern due to potential off-target editing. Several engineered SpCas9 variants specifically reduce off-target editing (Kleinstiver et al., 2016; Slaymaker et al., 2016; Chen et al., 2017). These variants are likely to be more clinically useful. Other recent studies suggested that CRISPR/Cas9 genome editing could select for cells with p53 mutations, a key driver of oncogenesis (Haapaniemi et al., 2018; Ihry et al., 2018). Similarly concerning were observations of off-target editing in human stem cells resulting from single nucleotide variants in the genome (Yang et al., 2014). Yet studies of rhesus monkeys, edited as embryos, discovered no off-target editing in functional genome regions (Chen et al., 2015; Wang S. et al., 2018). Similarly, large sequencing of genome edited mice, sheep, and goats has revealed very low off-target editing frequencies (Iyer et al., 2018; Li et al., 2018; Wang X. et al., 2018). Further studies, particularly with the engineered Cas9 variants, will be required to fully assess the rates of off-target editing in human primary cells.

An additional challenge is the limited animal models of HIV-1 disease. The possible immunogenicity of any foreign protein to a patient or animal may play a significant role in the success of the therapy (Crudele and Chamberlain, 2018). Several of these challenges, particularly complications due to the possible immunogenicity of Cas9, may be overcome in development of therapeutics for other diseases, such as diabetes (Wang et al., 2015, 2016c; Yin H. et al., 2016; Ratiu et al., 2017; Yin H. et al., 2017).

## Concluding Remarks

Instead of viewing genome editing of the HIV-1 provirus as a single cure therapy, this technology may be an additional approach in a combination therapy. For example, CRISPR/Cas9 vectors could be administered during continued ART. Alternatively, studies of CRISPR genome editing of the provirus in combination with siRNA have shown that these technologies may be additive in preventing HIV-1 replication in cell lines (Zhao et al., 2017). It is unlikely that any CRISPR/Cas9 gRNA regimen can be devised to target all quasispecies in a patient. However, additional investigation of PTCs may provide evidence that the intact proviral load can be decreased below a threshold that prevents viral rebound in the absence of ART. This is a highly speculative concept, but the possibility that a genome editing therapy could play a role in a functional cure of HIV-1 infection remains tantalizing.

## Author Contributions

All authors made intellectual contribution to this work and approved it for publication.

## Conflict of Interest Statement

The authors declare that the research was conducted in the absence of any commercial or financial relationships that could be construed as a potential conflict of interest.
